# P-TEFb- the final frontier

**DOI:** 10.1186/1747-1028-4-19

**Published:** 2009-09-02

**Authors:** Jiri Kohoutek

**Affiliations:** 1 Veterinary Research Institute, Hudcova 70, 621 00 Brno, Czech Republic

## Abstract

Regulation of gene expression is essential to all aspects of physiological processes in single-cell as well as multicellular organisms. It gives ultimately cells the ability to efficiently respond to extra- and intracellular stimuli participating in cell cycle, growth, differentiation and survival. Regulation of gene expression is executed primarily at the level of transcription of specific mRNAs by RNA polymerase II (RNAPII), typically in several distinct phases. Among them, transcription elongation is positively regulated by the positive transcription elongation factor b (P-TEFb), consisting of CDK9 and cyclin T1, T2 or K. P-TEFb enables transition from abortive to productive transcription elongation by phosphorylating carboxyl-terminal domain (CTD) in RNAPII and negative transcription elongation factors. Over the years, we have learned a great deal about molecular composition of P-TEFb complexes, their assembly and their role in transcription of specific genes, but function of P-TEFb in other physiological processes was not apparent until just recently. In light of emerging discoveries connecting P-TEFb to regulation of cell cycle, development and several diseases, I would like to discuss these observations as well as future perspectives.

## Introduction

Gene expression is a highly organized and tightly controlled process involved in a broad spectrum of biological processes, ultimately giving cells the ability to take control of their growth, cell division, differentiation and apoptosis. Regulation of gene expression is executed primarily at the level of transcription of specific mRNAs by RNA polymerase II (RNAPII), typically in several distinct phases: preinitiation, initiation, promoter clearance, elongation, RNA processing, and termination [1-3]. RNAPII is characteristic by the presence of an extended carboxyl-terminal domain (CTD), consisting of 52 tandem hepta-peptide repeats of canonical sequence, YSPTSPS [4]. Coincidently, CTD is subjected to numerous modifications, which control its ability to associate with transcription factors involved in RNA processing, elongation and termination [5]. Therefore, it seems that modification status of CTD is important for control of a particular phase of transcription [6].

Importantly, RNAPII can not initiate transcription alone. General transcription factors assist RNAPII to form a preinitiation complex (PIC) at the promoter of protein-coding genes. To initiate transcription, activity of TFIIF (CDK7/cyclin H) factor is required to begin promoter clearance and synthesis of short nascent RNA by RNAPII. TFIIH also phosphorylates Ser 5 at the CTD evoking its conformational changes that allow binding of capping complex and co-transcriptional capping of nascent RNA. After synthesizing around 50 ribonucleotides, RNAPII is recognized by negative elongation factor (NELF) and DRB-sensitivity inducing factor (DSIF) causing its promoter-proximal pausing. To overcome inhibitory effect of NELF/DSIF factors and to initiate productive elongation, the positive transcription elongation factor b (P-TEFb) is subsequently recruited to the paused/poised RNAPII. After phosphorylation of RNA recognition motif-containing protein RD (a NELF-E component) and Spt5 (a subunit of human DSIF) by P-TEFb, NELF leaves RNAPII; however, DSIF stays there and becomes a positive elongation factor [7-9]. Most importantly, P-TEFb phosphorylates Ser 2 of CTD, increasing its affinity towards components of splicing and polyadenylation machineries [3,10,11].

Much of what we know about regulation of elongation phase has come from studies using ATP analog 5,6-dichloro-1-b-D-ribofuranosylbenzimidazole (DRB). Treatment of cells with DRB caused dramatic reduction in mRNA synthesis characteristic by production of short capped RNA transcripts, suggestive of block in the elongation phase [12,13]. DRB blocked CTD phosphorylation as well [14]. Importantly, inhibition of RNAPII elongation by Flavopiridol, a selective P-TEFb inhibitor, resulted in abortive transcription of most protein coding genes [15].

It has been thought for years that formation of a preinitiation complex and subsequent recruitment of RNAPII to the promoter is a rate-limiting step in transcription regulation [16]. However, transcription of several genes, such as c-myc, HSP70, JunB, did not fit the general concept [17-20]. Over the years, it became increasingly clear that a block of the elongation phase is a critical control mechanism of transcription [21,22].

We have learned a great deal of P-TEFb genetics, biochemistry and molecular function from studying its function in HIV replication in cells. Nevertheless, function of P-TEFb in other physiological processes, such as cell differentiation, cell cycle, development and diseases, has not been pursued efficiently until just recently. The next parts of this review are dedicated to shed a light on new discoveries in these processes with future perspectives.

## P-TEFb - history and presence

P-TEFb is a heterodimer consisting of cyclin-dependent kinase 9 and one of the C-type cyclins T1, T2a, T2b or K [4,23-27]. CDK9 was first discovered by the Giordano lab as a cell division cycle 2 - related kinase with PITALRE motif [24]. It consists of 372 amino acids with a relative molecular mass around 42 kDa. It was believed that CDK9 42 kDa form (CDK9 or CDK9_42_) is the only functional form in cells. But such a presumption was challenged by a rather puzzling observation made by several laboratories. When commercially available antibodies specific for C-terminus or other domains of human CDK9_42 _were used in western blotting, an extra band migrating around 55 kDa was always detected [28,29]. Indeed, an additional form of CDK9 was identified in 2003 by the Price lab [29]. Transcription of this CDK9 form starts from an alternative TATA box upstream of previously described housekeeping-type promoter for the CDK9_42 _gene. Newly described form of CDK9 contains an entire amino acid sequence of CDK9_42 _and additional 117 amino acids extension bearing proline-rich region and glycine-rich region in its N-terminus [29,30]. The expression of both isoforms varies across different mouse tissues [30]. The CDK9_42 _is predominantly expressed in spleen, thymus and testes, whereas CDK9_55 _is highly abundant in brain, lung, spleen and thymus [30]. In many other cell types, the relative abundance of both isoforms depends on the original tissue, developmental stage, and cell commitment. For instance, CDK9_42 _isoform is highly expressed in human cervical carcinoma cells (HeLa) and mouse fibroblasts (NIH 3T3)[29]. An opposite picture is seen in primary hepatocytes, cultured macrophages or primary lymphocytes- in these cells, CDK9_55 _form is prevalent at steady state [29,30]. Yet, in hepatocyte cultures, in activated macrophages by lipopolysaccharide or lymphocytes by PMA/PHA CDK9_42 _is induced, while the level of CDK9_55 _remained relatively constant or decreases upon activation [29-31]. Interestingly enough, the CDK9_55 _form is almost undetectable in primary human monocytes or primary satellite cells, but its expression is robustly induced upon their differentiation, simply implying an essential function for CDK9_55 _in cell commitment and differentiation [31,32].

It is possible to speculate at the moment that the presence of an extra N-terminus in CDK9_55 _will play - at least in part - a key role in the final outcome of its function. Therefore, both forms of CDK9 might control transcription of diverse sets of genes and consequently be expressed in developmental or cell fate specific manner. For example, both CDK9 forms associate with MyoD, a factor involved in muscle differentiation, but CDK9_55 _seems to be more important for satellite differentiation during injury [32].

Four years later after discovery of CDK9_42_, a new type of cyclins, called C-type cyclins, was identified to associate with CDK9_42 _[26,27]. Cyclin T1 (CycT1) and two splicing isoforms of cyclin T2a and T2b (CycT2a and CycT2b) constitute the family of C-type cyclins. All cyclins associate with both forms of CDK9 with kinase activity towards CTD in RNAPII. They bear two prototypical cyclin boxes at the N-terminus, histidine-rich region, providing binding to CTD of RNAPII, and proline-serine rich C-terminus. CycT1, in contrast to CycT2a/b, contains TAR recognition motif (TRM) next to cyclin boxes, which is important for the formation of ternary complex between Tat/TAR/P-TEFb and initiation of HIV transcription [27,33]. Vice-versa, CycT2 bears a leucine-rich stretch next to its cyclin boxes capable of binding to CTD, thus providing an extra domain capable of targeting RNAPII [26,34]. Even though CycT1 seems to be the most abundant partner of CDK9 in cultured cell lines, the expression of both CycT2 isoforms is found in other cells and tissues as well [35,36]. Expression of CycT1/2 is regulated not just at the transcription level, but also at the level of RNA stability, translation and ubiquitination [37,38]. Several years after identification of CycT1 and CycT2, an additional cyclin was identified to associate with CDK9 by two hybrid screens of lymphocyte cDNA library [23]. This new member of C-type cyclins was called cyclin K and in contrast to CycT1 and CycT2, its whole C-terminus (normally found in other C-type cyclins) was missing. Three labs have shown that CycK can support phosphorylation of CTD by CDK9 *in vitro*, activation of P-TEFb-dependent genes when artificially tethered to RNA but not DNA, supporting its function in transcriptional elongation [23,25,39]. Moreover, expression of CycK is transcriptionally activated by p53, thus CycK could participate in control of cell cycle or apoptosis, but these observations were not followed in greater details [40].

Another milestone in P-TEFb biology was reached when two labs independently demonstrated that 7SK small nuclear RNA (7SK snRNA) is bound to P-TEFb and inhibits its kinase activity [41,42]. When RNase treatment or extracellular stress signals were used, active P-TEFb was released from association with 7SK snRNA, and transcription of long transcripts was restored. Surprisingly, later in 2003, the same laboratories discovered a protein which was able to cooperate with 7SK snRNA to inactivate P-TEFb in the 7SK small nuclear ribonucleic acid particle (7SK snRNP) or simply 'large' complex [43,44]. The protein was named hexamethylene bisacetamide inducible protein 1 (Hexim1), since Hexim1 was originally discovered as a protein induced in vascular smooth muscle cell after exposure to hexamethylene bisacetamide (HMBA) [45]. Later the same year, Hexim2 was discovered as an additional member of the Hexim protein family. It is highly homologous to Hexim1 and was shown to substitute function of Hexim1 *in vivo *and *in vitro *[46,47]. Importantly, Hexim1 and Hexim2 can form homo- or heterodimers to incorporate P-TEFb into the large complex. The oligomerization is mediated through the basic region within the central part, with bound 7SK snRNA, and its coiled-coil region in the C-terminal domain [48,49]. The binding of 7SK snRNA to the basic region in the Hexim oligomer induces exposure of the CycT1-binding domain in its C-terminus and formation of large complex [43,44,46-50]. Hexim1 and Hexim2 do not represent bona fide cyclin-dependent kinase inhibitors (CKI). The most surprising feature of both is the fact that they associate with 7SK snRNA first in order to inhibit P-TEFb; therefore, they exhibit a completely new group of CKIs, since none of the so-far studied CKIs utilize RNA as a partner to inhibit kinase activity of CDK/cyclin complex.

Critically, the exploration of P-TEFb associating partners in the large P-TEFb complex has not been finished yet. Two additional proteins participating in the formation of large P-TEFb complex were identified during the last two years. MEPCE (7SK snRNA methylphosphate capping enzyme) was identified as a specific 7SK snRNA methyltransferase causing methylation of the gamma-phosphate of its first 5' nucleotide [51]. LARP7 (La-related protein 7) stabilizes 7SK snRNA by binding to its 3'-UUUU-OH tail protecting it from degradation by exonucleases [52-54]. Both proteins are stably associated with 7SK snRNA after release of P-TEFb caused by stress or cellular signals in contrast to Hexim1, which are displaced from 7SK snRNA right after disruption of large complex [52-56].

**Figure 1:**
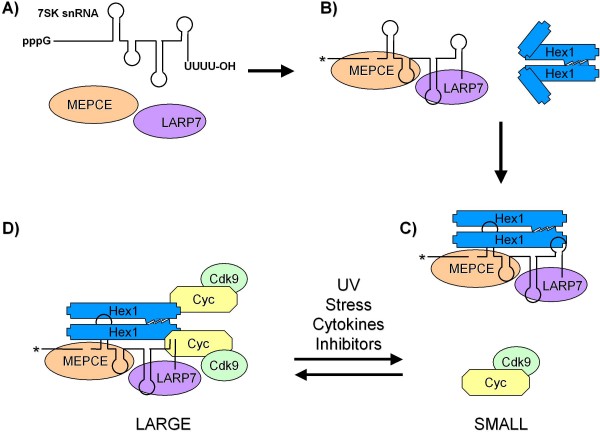
**Composition and assembly of P-TEFb complexes**. **A) **7SK snRNA contains 5'- and 3'- ends with pppG (triphosphate guanosine) and UUUU-OH (oligouridylate tail), respectively. 7SKsnRNA is recognized by MEPCE (7SK snRNA methylphosphate capping enzyme) and LARP7 (La-related protein 7). **B) **MEPCE methylates gamma-phosphate of its first 5'ribonucleotide, depicted by an asterisk, and LARP7 stabilizes 7SK snRNA by binding to its oligouridylate tail. Hexim1 (Hex1) homodimerizes (or heterodimerizes with Hexim2) via its coiled-coil domain in the C-terminus, but N-terminus adopts conformation which does not allow binding to P-TEFb (CDK9/Cyc). **C) **Binding of 7SK snRNA from 7SK snRNA/MEPCE/LARP7 complex to basic region in central part of Hexim1 triggers conformational changes of hexim dimer leading to exposition of CycT1-binding domain at the C-terminus of Hex1 and consequent binding of P-TEFb ('SMALL' complex). D) The 'LARGE' complex is formed and is stabilized due to multiple protein-protein and protein-RNA contacts within the complex. Activity of P-TEFb is inhibited in the large complex. Several stimuli have been reported to disrupt the large complex, such as UV radiation, diverse stress signals (mechanical, hypertrophic), cytokines (TNF-α, IL-6) and inhibitors (Actinomycin D, DRB).

Importantly, studies of biology of active complex led to identification of bromodomain protein 4 (Brd4), a major binding partner of P-TEFb when 7SK snRNA along with Hexim1/2 are displaced from P-TEFb [57,58]. Brd4 binds to acetylated histones and might be therefore targeting P-TEFb to actively transcribed genes if there is no specific factor recruiting P-TEFb to these genes [59]. Therefore, P-TEFb is typically present in two distinct complexes in most cell types. Heterodimer of CDK9/cyclin represents active P-TEFb and is here referred to as a 'small' complex of P-TEFb, irrespective of Brd4 binding (Figure 1). Whereas, cooperative binding of P-TEFb/cyclin/7SK snRNA/Hexim1 or 2/MEPCE/LARP7 identifies an inactive P-TEFb form, also recognized as 'large' complex of P-TEFb (Figure 1). Application of stress stimuli, UV radiation, cytokine treatment, chemical compounds, etc. on cells leads to dissociation of P-TEFb from Hexim1/2 and 7SK snRNA/MEPCE/LARP7 (Figure 1).

## Posttranslational modifications of P-TEFb components

Besides inhibition of P-TEFb by recruitment to the large complex, its activation or inhibition depends primarily on posttranslational modifications of CDK9 and its associated cyclin. We would like to discuss relevance of these modifications in respect to their putative functions in development. During many developmental processes, such as gastrulation, left-right patterning, organogenesis and many others, the morphogens are literally a moving force navigating development forward. Morphogens can make appropriate impact only if there is a proper receptor on cell surface, which can pass message through various cellular signaling pathways to particular transcription activators or repressors. Thus, it is highly possible that the same signaling circuits involved in signal transmission could target and consequently modify activity of P-TEFb either in a positive or negative way.

Among so-far documented posttranslational modifications of P-TEFb, phosphorylation of cyclin and especially of CDK9 is a key feature of its regulation *in vivo*. Several serines and threonines residues (Ser347, 354 and 357; Thr350 and 354) at its C-terminus must be phosphorylated for P-TEFb activity first [60,61]. Nevertheless, full activation of P-TEFb is completed after conserved Thr186 in the T-loop is phosphorylated, an event triggering major conformational changes in CDK9/CycT1 heterodimer leading to exposition of ATP binding pocket together with substrate site [49,62,63]. Recently, phosphorylation of Thr29 in CDK9_42 _within the HIV elongation complex was able to block transcription of viral RNAs [64]. To complete list of CDK9/cyclin phosphorylation status, several other residues, not previously described, were identified by global mass spectrometric technology to be phosphorylated *in vivo *[65,66]. Of course, functional consequences of these modifications have not been investigated yet, but they might serve as a first clue for further studies to identify appropriate kinases.

Phosphorylation of Thr186 is also a prerequisite for the assembly of the large complex [49,62]. The reason why large complex bears principally active P-TEFb is most likely to allow cells, in time of need, an efficient and fast release of stored P-TEFb to support transcription of genes involved in given cellular responses. Indeed, certain stress or pathological conditions induces liberation of P-TEFb from the large complex. Two years ago, PI3K/Akt signaling pathway was reported to disrupt large complex, by phosphorylating Hexim1 on two serines and two threonines in the CycT-binding domain, after HMBA treatment [67]. In addition, the Zhou lab demonstrated that UV and HMBA, agents known to activate P-TEFb, disrupt P-TEFb from the large complex by cooperative action of calcium ion (Ca2+)-calmodulin-protein phosphatase 2B (PP2B) and protein phosphatase 1 (PP1α) [68]. In detail, activated PP2B provokes conformational changes in 7SK snRNP first, allowing PP1α to liberate P-TEFb by dephosphorylating Thr186. Alternatively, PPM1A and PPM1B (protein phosphatases, magnesium-dependent) were found to mediate dephosphorylation of Thr186 in cells under non-stress conditions, pointing towards several alternative mechanisms used by cells to accommodate different stress or non-stress (physiological) stimuli [69]. Nevertheless, inactive P-TEFb (dephosphorylated on Thr186) is subsequently recruited to the transcription initiation complex by Brd4. These finding correlates with *in vitro *studies demonstrating that Thr186 is kept unphosphorylated by action of TFIIH in the HIV preinitiation complex [70]. Interestingly, phosphorylation of Thr29 in CDK9 mediated by Brd4 was detected in HIV transcription initiation complex just recently [64]. Upon dissociation of TFIIH and Brd4 during elongation transcription induced by Tat, P-TEFb is fully activated by *de novo *phosphorylation of Thr186 and dephosphorylation of Thr29 [64,70]. Given importance of protein phosphatase-2A (PP2A) in the augmentation of basal activity of the HIV-1, it is tempting to speculate that PP2A is a phosphatase acting on Thr29 [71].

Even though phosphorylation might seem to be most essential for P-TEFb activity, ubiquitination and acetylation participate in regulation of P-TEFb activity as well. Ubiquitination of CycT1 and CDK9 by Skp2 controls its protein turnover and interaction with Tat/TAR, respectively [72,73]. Also, human double minute-2 protein (HDM2), a p53-specific E3 ubiquitin ligase, ubiquitinates Hexim1 in the basic region [74]. Ubiquitination of Hexim1 is not involved in Hexim1 proteasome-mediated protein degradation but rather interferes with inhibition of P-TEFb [74]. Further, acetylation of CDK9 on Lys44 located in the ATP binding domain by p300/CBP increases its kinase activity towards CTD of RNAPII [75]. In contrast, acetylation of two lysines at positions 44 and 48 in CDK9 by P/CAF and GCN5 complexes exhibited inhibition of its kinase activity and relocalization to insoluble nuclear matrix [76]. Importantly, acetylation of cyclin T1 triggers dissociation of P-TEFb from the large complex and its activation [77]. Given the importance of p300/CBP in the acetylation of Tat and activation of HIV transcription, it is not surprising that the HIV virus utilizes the same acetyl transferase complex to acetylate Tat in order to stabilize formation of Tat-P-TEFb-TAR complex to initiate viral transcription [78]. Therefore, one could assume that the virus intentionally subverts natural cellular cofactors of P-TEFb to support its own replication cycle.

Together documented posttranslational modifications are of great interest in respect to their possible function in regulation of P-TEFb activity in diverse cellular responses, (stress, cytokines, cell communication), disease and development. One could predict that simple recruitment of P-TEFb to the promoter of given genes does not guarantee transcriptional initiation *per se*. Rather, current pattern of posttranslational modifications (phosphorylation, acetylation, ubiquitination) of subunits of P-TEFb will decide if transcription is turned on or off. For example, two P-TEFb complexes located at two different promoters might differ in transcriptional response to given signals, because of the Thr29 phosphorylation or Lys44 and 48 acetylation of CDK9 within one of the P-TEFb complexes.

## Function of P-TEFb in cell cycle

Each phase or intercalating transitions of cell cycle are controlled by spatio-temporal expression of CDKs and appropriate cyclins, but in the case of CDK9 and C-type cyclins, neither levels nor associating CDK9 kinase activity were changed or dramatically fluctuated during this process [24]. Such an observation does not necessary rule out CDK9 function in the control of cell cycle, since protein level or associated kinase activity of other CKDs and cyclins are not changing during cell cycle as well [79]. Nevertheless, recent identification of Brd4 can provide the missing link to the function of P-TEFb in cell cycle control, since Brd4 is implicated in transition of epigenetic memory through binding to acetylated histones [57-59,80]. The Zhou lab was able to demonstrate a dramatic increase in P-TEFb-Brd4 interaction from late mitosis to early G1 phases of cell cycle and active recruitment of P-TEFb to the chromosomes, followed by initiation of transcription of key genes for G1 progression. Importantly, depletion of Brd4 abrogated the whole process by reducing transcription of essential G1 genes, leading to G1 cell cycle arrest and apoptosis [80]. Therefore, it is very tempting to speculate that Brd4-mediated recruitment of P-TEFb to G1 genes is fundamental for successful G1/S transition and could serve as a hallmark of transcriptional memory across cell division [59,80]. However, other functional aspect/s of Brd4-P-TEFb interaction in respect to cell cycle must be explored first. For instance, how is the association of Brd4 with P-TEFb and consequent recruitment to specific genes regulated in cell cycle-dependent manner in the first place? From recent studies, we have learned that Brd4 recruits P-TEFb to inflammatory and Hox genes by interaction with acetylated NF-κB on Lys-310 or acetylated histone 4 on Lys-5, 8 and 12, respectively [81,82]. Also, acetylation of histone 3 at promoter-proximal regions in CD80 genes was critical for Brd4-dependent P-TEFb recruitment and transcription initiation [83]. If the same mechanisms/principles apply for Brd-4/P-TEFb recruitment to chromosomes at G1 phase remains to be explored in the future.

Moreover - is simple recruitment of P-TEFb to Brd4 enough or are posttranslational modifications involved too? If the answer is yes, then what residues are involved, and most importantly, what is their nature (phosphorylation, acetylation, etc)? Such a scenario would imply an existence of specific complexes able to carry these modifications. What are these factors? It will be of great interest to explore in more detail if CDKs participating in M or G1 phases are engaged in these events, but involvement of other protein kinases must be taken in account, too.

Additional function of P-TEFb in cell cycle could be in connection to the retinoblastoma protein (pRB). CDK9 was first identified as a cell division cycle-2 related kinase, with strong kinase activity towards pRB, but not histone 1, suggesting its role in G1/S transition [84]. Indeed, silencing of CDK9 in cells by RNA interference approach led to the enrichment of cells in G1 phase of cell cycle supporting function of P-TEFb in the G1/S transition [85]. Interestingly, CDK9/CycT2 but not CDK9/CycT1 complex binds pRB suggesting diverse function of C-type cyclins in cell cycle regulation [86].

Last but not least, CDK9 phosphorylates p53 on multiple Ser residues (Ser33, 315 1nd 392). Phosphorylation of Ser 33 and 315 was implicated in the association with propyl isomerase Pin1. Phosphorylation-dependent Pin1/p53 interaction induces conformational changes in p53 leading to elevated DNA binding and transactivation capacity [87]. Phosphorylation of 392 resulted in stabilization of p53 tetramer and enhancement of target gene expression [88,89]. Interestingly, Cdk9 gene was also activated by binding of p53 to its promoter, suggesting a positive role of p53 in the regulation of its basal transcription [88]. Since Ser392 phosphorylation is not required for p53-mediated cell cycle arrest, it is possible to speculate that there is a positive regulatory loop between expression of CDK9 and p53 transactivation mediated by Ser392. Nevertheless, two major questions need to be elucidated. First, why does CDK9 phosphorylate p53: is it to strengthen DNA repair machinery or is to support apoptosis mediated by p53? Second, why does p53 need to control expression of CDK9, is it required for its function or is it to ensure optimal expression of other genes involved in adequate p53 response?

## Function of P-TEFb in development

In the past decade, we have learned a great deal about genetic, biochemical and molecular properties of P-TEFb, mostly due to its indispensable role in HIV replication, cytokine responses, cell differentiation, etc. Just very recently we have started to appreciate physiological role/s of P-TEFb in development. Individual genetic depletion of CycT1 or CycT2 in Caenorabditis elegans had no dramatic impact on its development. In contrast, genetic inactivation of both cyclins simultaneously resulted in early embryonic lethality, similarly to genetic inactivation of RNAPII [90].

To test whether P-TEFb is essential for Drosophila development, CDK9 was down-regulated by RNA interference (RNAi) approach. CDK9 knock-down flies died during metamorphosis, suggesting fundamental role of P-TEFb in Drosophila early development [91]. Relatively later lethality in flies in comparison to C. elegans could reflect major differences between these two species. First, timing and efficiency of CDK9 knock-down could differ and most importantly, stored maternal mRNA or protein might compensate for the loss of *de novo *expression of CDK9 for some time [91].

The role of CDK9 in zebra fish development was investigated by usage of specific morpholinos (MO), which mediate degradation of CDK9 messenger RNA and consequent down-regulation of CDK9 protein. Injection of specific Cdk9-morpholinos had a severe effect on definitive erythropoiesis manifested by significant reduction of runx1 signal in the dorsal aorta precursor population [92,93]. Taking into account ubiquitous expression of CDK9 in the whole developing embryo, it is surprising that only an effect on hematopoietic system was observed. It was probably due to insufficient depletion of CDK9 from other cells; therefore, these cells have enough CDK9 for their proper function. In the case of hematopoietic cells, the level of CDK9 did not reach critical threshold necessary for terminal differentiation of hematopoietic precursors. Supporting evidence to this threshold scenario comes from studies of HIV replication in cells, too. RNA mediated knock-down of P-TEFb in HeLa cells did not cause cellular death but inhibited Tat transactivation and HIV-1 transcription instead [94].

To investigate final consequences of nonfunctional P-TEFb in mice, we tried to generate knock-out mice for CycT1 and CycT2 using the β-geo gene trap technology [95]. Regretfully, our attempts to generate a complete knock-out mouse for CycT1 were not successful, and only a hypomorphic mouse with residual expression of CycT1 in the whole body was generated. These hypomorphic mice exhibited only modest immunological defects, such as, altered class switch recombination (not published data) and moderate appearance of autoimmunity due to impaired negative selection of autoreactive T cells in thymus [96]. In the case of CycT2, after conducting numerous matings of heterozygous mice, we were not able to detect any newborn mice bearing nonfunctional allele of *CcnT2 *gene. When developing embryos and fetuses at different developmental stages were genotyped, still no null animal for *CcnT2 *gene was found, suggesting a developmental block even before mid gestational period. Indeed, embryonic lethality preceding even blastocyst implantation was observed. This early lethality could be attributed to impaired zygotic gene activation taking place in two-four cell embryos or to decreased expression of critical genes, which were revealed by siRNAs against CycT2 in embryonic stem cells [36,97].

So far, only examples of developmental consequences of P-TEFb loss of function were described, but ectopic activation of P-TEFb exhibits dramatic consequences for normal development as well. Genetic ablation of Clp-1, the mouse homologue of human Hexim1, resulted in embryonic death at E16.5 due to cardiac hypertrophy [98]. Down-regulation of LARP7 orthologue in zebra fish caused embryonic death due to aberrant splicing, suggesting a fundamental role for P-TEFb in coupling transcription elongation with alternative splicing [52].

To finalize developmental properties of P-TEFb, we would like to describe a situation where inhibition of P-TEFb is necessary indeed for normal development of germ-line blastomeres (C. elegans) or polar cells (D. melanogaster), specialized cell types similar to primordial germ cells in mammals. These cells are kept in undifferentiated stage by repressed mRNA transcription due to absence of Ser2 phosphorylation in CTD of the RNAPII. PIE-1 in C. elegans and pgc (polar granule component) in D. melanogaster block Ser2 phosphorylation by binding to P-TEFb and preventing its recruitment to CTD [99-101]. Predictably, the next step should be to elucidate if the inhibition of P-TEFb is a common mechanism for germ line specification in other species as well. Similarly to PIE-1 and Pgc, Runx1, a repressor of CD4 expression in double negative thymocytes, disables transcription of CD4 gene by decoying P-TEFb from the already engaged RNAPII [102].

Collectively, we have learned a key role of P-TEFb in early development from nematodes to mammals, but still we do not know what the target genes are within developmental programs. The first clue pointing to the right direction/s could come from our siRNA experiments in ES cells [36]. RNAi approach was used to down-regulate CycT1 or CycT2 and check for changes, by microarrays, in the expression of genes influenced by depletion of either cyclin. Reduction in CycT2 affected mostly expression of genes participating in the TGFβ and Wnt signaling pathways, as well as autophagy. The most affected genes were Lefty 1 and Lefty 2, members of the TGFb superfamily, which are highly expressed in the inner cell mass and trophoectoderm during embryogenesis [103]. Moreover, genes involved in ubiquitin-proteasomal system and autophagy, which are indispensable for rapid degradation of maternal protein during the transition from oocytes to embryos, were also lessened. Of note, autophagy-defective oocytes fail to develop beyond the four- and eight-cell stage [104]. In contrast, CycT1 knock-down decreased expression of genes involved in fatty acid and glucose metabolism, cell communication and cell cycle [36]. Critically, although glucose metabolism was affected in both cases, targeted genes were different.

From these or similar microarray data, we can indeed pinpoint target genes of different P-TEFb complexes, yet fundamental questions remain to be solved.

- 1) ***How does P-TEFb achieve its broad binding capacity towards myriad transcription factors?***

- 2) ***What are principal transcription factors driving normal development, cell fate commitment or terminal differentiation through interaction with P-TEFb?***

- 3) ***Why is only a specific subset of P-TEFb-dependent genes transcribed in response to given intra- and extracellular stimuli?***

**Figure 2:**
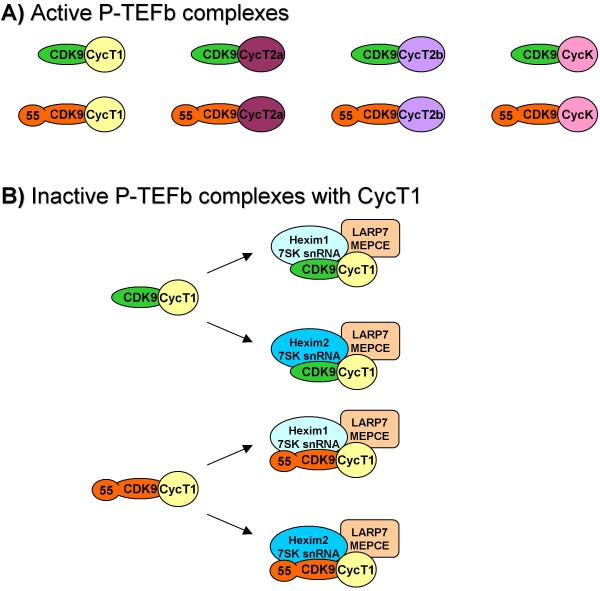
**Active and inactive P-TEFb complexes**. **A) **Active P-TEFb complexes. CDK9_42 _(green oval) and CDK9_55 _(orange oval) can separately bind to individual CycT1 (yellow circle), CycT2a (violet oval), CycT2b (lavender oval) and CycK (pink oval). **B) **Inactive P-TEFb complexes with CycT1. Only large complexes with CycT1 are presented for illustration, but the same would apply for CycT2a, CycT2b and CycK too. Complexes of 42- and CDK9_55 _with CycT1 are presented at the left side. 'Large' complexes consisting of CDK9/CycT1 are at the right side. Hexim1/7SK snRNA (light blue oval), Hexim2/7SK snRNA (turquoise blue oval), MEPCE/LARP7 (light orange oval).

***Ad 1*) **Usually, when we think of P-TEFb, two functional states are considered: active (small complex, typically CDK9_42_/CycT1) and inactive (large complex, CDK9_42_/CycT1/7SK snRNA/Hexim1/LARP7/MEPCE). One must actually revise our rather simplified view on P-TEF complexes, as proposed in several publications [36,105]. Active P-TEFb consists of CDK9 and C-type cyclin, but two forms of CDK9 exist (CDK9_42 _and CDK9_55_) and at least four isoforms of C-type cyclins (CycT1, CycT2a, CycT2b and CycK). By simple combinatorial math, it leaves us with 8 different complexes of active P-TEFb (Figure 2A). Taking in account the existence of Hexim1, Hexim2, 7SK snRNA, LARP7 and MEPCE, we will come to number 16 for inactive P-TEFb complexes (Figure 2B). All together there are 24 P-TEFb complexes with unique molecular surface composition. One might argue that binding of P-TEFb strictly depends on recognition capacity of cyclin boxes, histidine-rich and leucine rich regions in cyclins and substrate binding site in CDK9, but other possibilities should be considered, too. CDK9/cyclin adopts more open conformation different from CDK2/CycA providing extra molecular surfaces available for new interactions [63]. Also, other components of small or large complexes can mediate interaction with various factors. Indeed, nucleophosmin and NFkB associate with basic region of Hexim1 [45,106]. Also, estrogen and glucocorticoid receptors bind Hexim1 through its basic region [107,108]. Examples of these associations have served only to demonstrate hidden reserves of P-TEFb to interact with distinct factors.

***Ad 2*) **We found that Lefty1 and Lefty2 proteins were less expressed in ESc with down-regulated CycT2 but not CycT1. Oct4, Sox2, key transcriptional factors guarding self-renewal property of ES, were shown to bind to the promoter of Lefty1 gene and activate it [109]. Thus, it is possible that CycT2 binds specifically Oct4 and Sox2 but CycT1 does not. Along with this line, CycT2 together with CDK9_42 _is required for myogenesis *in vitro *by activating MyoD-dependent transcription [110]. On the contrary, MEF-2 related to MyoD, associates with CycT1 [111]. Recently, function of P-TEFb complex composed of CDK9_55 _and CycT2 was demonstrated to play a fundamental role in muscle regeneration by governing myoblast differentiation from satellite cells *in vivo *[32]. These examples demonstrate clearly that one developmental program (myogenic differentiation) can employ three distinct P-TEFb complexes to regulate a particular part of differentiation process. Of note, two repressors of MyoD family, I-mfa (inhibitor of MyoD family) and HIC (human I-mfa-domain-containing) employ P-TEFb for their function in myogenic differentiation too [112]. In addition, PPARγ (peroxisome proliferator-activated receptor γ), a master regulator of adipogenesis, utilizes CDK9_55 _for its function [113]. Importantly, CycT2 was identified by two-hybrid screen as a Mix.3/mixer, a Pax-like homeodomain protein, essential for endoderm formation [114]. On the other hand, association of CycT1 with GATA-1, a transcription factor involved in differentiation of numerous hematopoietic lineages, is indispensable for efficient megakaryopoiesis [115]. Last but not least, CDK9 participates in haematopoiesis in zebra fish through regulation of Ldb1 (LIM domain binding protein), which controls transcription of Hox genes [93]. Thus, specific transcription factors operating in certain developmental process utilize different P-TEFb complexes for their developmental tasks.

***Ad 3*) **We believe that two equally important mechanisms help P-TEFb to decide if certain gene/s will be turned on or off. A) One layer of control is provided by functional interaction with specific cofactors (chromatin remodeling complexes, mediators) bound at transcription sites. B) The other one is the result of co-operative effect of signaling pathways regulating P-TEFb activity.

**Figure 3:**
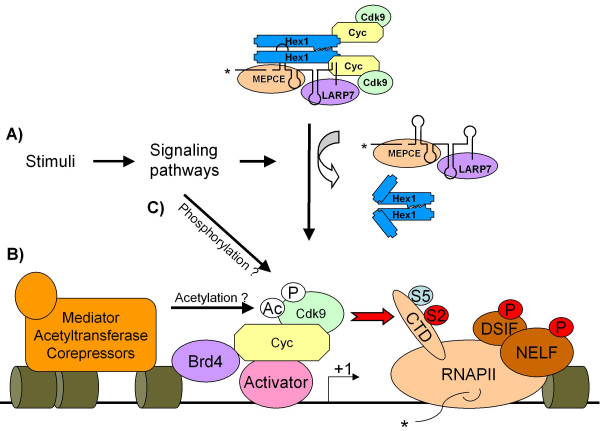
**Regulation of P-TEFb activity during transcription**. **A) **P-TEFb is inactivated in the large complex. After given stimuli (UV radition, cytokines, hypertrophic signals), active P-TEFb is released from the large complex by activity of signaling pathways downstream of these stimuli. Then, P-TEFb is recruited to the responsive promoter/s either by a specific activator (pink oval) such as MyoD or a co-activator, such as Brd4 (violet oval). P-TEFb is engaged at the promoters with poised RNAPII phosphorylated on Ser5 (light blue oval with letter S5) in CTD mediated by TFIIH. P-TEFb initiates transcription elongation by phosphorylating Ser2 of CTD in RNAPII (red oval with letter S2), DSIF or NELF (red ovals with letter P). Still, activity of P-TEFb can be controlled by two additional mechanisms. **B) **If there is a co-factor (acetyltransferase, mediator, corepressor) associating with P-TEFb at the promoter, then the activity of P-TEFb will depend on particular posttranslational modifications mediated by these co-factors, for example acetylation of Lys44 and 48 in CDK9 (white oval with letters Ac). **C) **Also, signaling pathways activated by given stimuli can modify components of the small complex, for example phosphorylation of CDK9 on Thr28 and Thr186 (white oval with letter P), and additionally modulate final activity of P-TEFb. Khaki barrels represent nucleosomes, and +1 depicts transcription start site.

**- A) **Once P-TEFb is recruited to the promoter, it is exposed to interactions with other multiprotein complexes and to their catalytic activities resulting in posttranslational modifications triggering activation or inhibition of P-TEFb activity (Figure 3B). Indeed, representatives of histone acetyltransferases p300/CBP and GCN5 with P/CAF can acetylate CDK9 to either activate or repress its kinase activity [75,76]. BRG1, a component of mammalian homologous of the SWI/SNF chromatin remodeling complexes, binds CDK9; together they activate STAT3-mediated transcription in response to cytokine receptor stimulation [116]. Moreover, co-repressor of thyroid hormone receptors (TR) and retinoic acid receptors (RAR) - N-CoR - associates with Hexim1 affecting ability of CDK9 to phosphorylate CTD of RNAPII [75].

**- B) **Signaling pathways serve, most likely, two primary functions in P-TEFb biology: first to trigger release of P-TEFb from the large complex; second to target and modify components of P-TEFb complexes (Figure 3A and 3C). At present, we know what stimuli/signals dissociate P-TEFb from the large complex, but we have just begun to explore signaling pathways responsible for it (see also posttranslational modification). Regretfully, we have almost no basic knowledge about signaling pathways operating and targeting P-TEFb activity during development, except for few examples. The PKNα, a fatty acid- and Rho-activated serine/threonine protein kinase, which was shown to bind CycT2a and consequently enhanced CycT2a mediated expression of myogenic differentiation markers during starvation, induced differentiation [117]. Further, the MEK1-extracellular signal-regulated kinase (ERK) signaling pathway promotes CDK9/CycT1 dimer formation and induction of immediate early genes (like c-*fos*) in neuroendocrine cells [118]. From our microarray data we know that transcription of Lefty1 is CycT2-dependent [36]. Lefty1 contributes to the establishment of left-right symmetry during vertebrate development. Bone morphogenetic protein (BMP) type I receptor was demonstrated to positively regulate Lefty1 expression in the chimeric embryo [119]. Therefore, one could hypothesize that BMP signaling could be involved in P-TEFb (CycT2) activation. Even if we identify pathways modulating P-TEFb activity, it is still critical to determine what component/s of P-TEFb complexes they target and what the nature of particular modification is. In summary, it is realistic to assume that recruitment of particular P-TEFb complex in combination with transcriptional co-factors to unique set of genes can control various aspects of transcriptional programs in normal development.

Except already introduced transcriptional factors, a plethora of diverse transcriptional factors/enhancers were found during the past decade to utilize P-TEFb for various cellular pathways and physiological processes: 1) cytokine signaling - p65 subunit of NFκB, STAT3 [45,116,120,121]; 2) hormone nuclear receptors - estrogen, glucocorticoid and androgen receptors [107,122,123]; xenobiotic sensing - arylhydrocarbon receptor [124]; immunity - class II transactivator, AIRE [96,125,126] and cell proliferation/differentiation - c-myc [127]. P-TEFb plays a special function in HIV infection by initiating its transcription through binding to Tat, a specific viral transcriptional transactivator [27].

## Promoter-proximal pausing of RNAPII

At last, we would like to dedicate a special part of this review to a very intriguing function of P-TEFb in developmental control through activation of stalled RNAPII. Historically, initiation of transcription was viewed as a rate-limiting step in the expression of majority of genes [16]. Interestingly, early studies indicated that specific genes, such as c-myc, junB and HSP70, surpass this concept, and their expression is subjected to elongation control through poised polymerase [17,18,20]. In principal, RNAPII initiates transcription but is immediately poised after synthesizing short RNA by action of negative elongation factors at the promoter-proximal region [21]. Nevertheless, a growing body of evidence demonstrates that RNAPII pausing is a novel mode of transcription control rather than exception from the rule [128-130]. Recent studies in Drosophila and mammalian system validated RNAPII distribution across the whole genome and clearly demonstrated that a significant number of genes was regulated at an early step of transcription elongation [129,130]. Among them, genes involved in development, cellular response to stimuli, cell communication, cell adhesion and differentiation demonstrated promoter-proximal RNAPII stalling. Importantly, down-regulation of NELF by RNAi resulted in loss of stalled RNAPII mark and re-expression of most of these genes [129]. To extend the concept of stalled polymerase to developmental perspective, transcription of *Hox *genes, governing anterior-posterior patterning in metazoan embryos, was found to be regulated at the level of elongation. Intact CDK9 activity was necessary to alleviate stalled RNAPII at the promoter of two *hox *genes in Drosophila (Ultrabithorax and Abdominal-B) [131]. Authors also suggested that Cdk9 could be involved in the regulation of Notch-, EGF-, and Dpp-signaling genes, again pointing towards connection of P-TEFb and regulation of developmental genes [131].

During preparation of this manuscript, new publication had appeared focusing on biological function of stalled RNAPII in the signal-dependent gene expression [81]. To study genes fitting to this criterion, authors employed LPS-inducible inflammatory gene expression in macrophages to demonstrate: 1) that genes induced in the primary response (LPS) have stalled RNAPII at the promoter-proximal region, 2) their induction is regulated by signal-dependent P-TEFb, 3) which is recruited via Brd4 binding to Histone 4 acetylated on Lys 5, 8 and 12. Moreover, transcription of these genes is repressed before stimulation by cooperative function of repressors [81]. Interestingly, developmentally regulated genes share the same characteristics as the LPS-inducible genes. It is stalled RNAPII and hierarchical expression of activators/repressors along with their co-factors.

These are very important observations in respect to possible role of P-TEFb in development. Since P-TEFb induces a transition form abortive to productive transcription elongation by phosphorylation of NELF and DSIF, it is logical to envision that P-TEFb might be a primary sensor to various developmental demands. In theory, P-TEFb could work as a platform capable of accommodating diverse stimuli and respond to them by switching on and off appropriate set of genes, along with cooperative function of developmental activators and repressors. Considering all available facts, it is now becoming clear that elongation block, by means of stalled RNAPII, represents a highly specific control module in transcription regulation.

## P-TEFb and disease

### Cardiac hypertrophy

Cardiac hypertrophy is probably the best illustrative example how deregulation of p-TEFb activity manifests in pathological phenotype, a disease. Cardiac hypertophy (heart growth) is characterized by enlargement of cardiac myocytes in response to diverse signals, such as biomechanical stress, sarcomeric and cytoskeletal protein mutations, G protein-coupled receptors for ligands, etc. At the molecular level, cardiac hypertrophy is characterized by an increase in cell size and protein synthesis and reactivation of the fetal gene program [132]. In turn, increased synthesis of mRNA species results in elevated activity of RNAPII phosphorylated at Ser2 within CTD [133]. Ser2 is a target of P-TEFb, thus it is not surprising that P-TEFb was identified to be the limiting factor for pathological manifestation *in vitro *and *in vivo *[98,134]. Briefly, ectopic activation of P-TEFb either by ablation of cardiac lineage protein 1 (Clp-1), the mouse homologue of Hexim1, or overexpression of CycT1 in adult heart led to fetal lethality or heart growth due to cardiac hypertrophy, respectively [98,132].

Striking characteristic of cardiac hypertrophy is the fact that all hypertrophic stimuli led to release and activation of P-TEFb [132]. Since most of the signaling pathways downstream of these signals are well characterized, it will be critical to determine their role in the activation of P-TEFb. Truly enough, Jak/STAT signal transduction pathway is involved in the release of P-TEFb from large complexes [135]. Yet, it is not clear at the moment if Jak/STAT pathway targets and modifies any component of the large complex directly or indirectly. Moreover, from developmental perspectives of P-TEFb, it is highly probable that the signaling pathways governing pathological activation of P-TEFb will be, in part, the same signaling circuits driving growth of heart earlier during normal heart development [135]. Therefore, it will be of great interest to explore and characterize what the major differences in signaling pathways diverging to either adaptive or pathological P-TEFb activation are. In particular, what are these pathways by nature? Do they 'just' disrupt large complex or do they modify components of large/small P-TEFb complexes? Finally, how can we use this acquired knowledge to modulate de-repressed activity of P-TEFb in various diseases?

### Cancer

Historically, the first recognition of P-TEFb in malignant processes came from a study focused on identification of novel tumor antigens associated with serous ovarian cancer [136]. By employing SEREX immunoscreening, the authors identified 9 immunogenic antigens, among them Hexim1, to be potential targets for immunotherapy. Relevance of P-TEFb in progression of ovarian cancer was not recognized at the time, since connection between Hexim1 and P-TEFb had not been established yet.

Another piece of the P-TEFb puzzle in cancer came from studies of mixed-lineage leukemia (MLL) fusion proteins in leukemic transformation. The gene for the histone methyltransferase MLL often participates in chromosomal translocations that eventually create MLL-fusion proteins associated with very aggressive forms of childhood acute leukemia [137,138]. Two proteins, Eleven Nineteen Leukemia (ENL) and AF4 proteins, common associating partners of MLL in childhood acute leukemia, were found to bind and utilize P-TEFb for their transformation properties [139,140]. These studies provided compelling evidence for direct role of AF4 and ENL in the regulation of transcription elongation and chromatin modification. This could also suggest that therapies targeting P-TEFb activity in leukemia might be a direction to pursue. Indeed, Flavopiridol, a specific inhibitor of CDK9, was able to induce apoptosis in chronic lymphocytic leukemia cells by suppressing transcription of short-lived antiapoptotic proteins, such as Mcl-1 [141].

Connection of Hexim1 in malignant processes through its binding to estrogen receptor was recently provided [108]. Estrogen receptors (ERs) are found in significant numbers of breast cancer, and targeted therapy against ERs has been used intensively [142]. Hexim1 binds ERα through its basic region and blocks ERα mediated gene expression [123]. Critically, overexpression of Hexim suppressed proliferation of breast cells. In accordance with this finding, expression of Hexim1 was down-regulated in samples from invasive breast cancer patients in comparison to Hexim1 level in normal breast tissue [108].

Certainly, the most illustrative example of P-TEFb dysfunction in malignant conversion has been demonstrated by the Zhou lab [53]. They identify PIP7S, also known as La-related protein 7 (LARP7), to associate and stabilize 7SK snRNP formation. LARP7 contains 3 RNA binding motifs, La binding motif in its N-terminus, a RNA recognition motif (RRM) 1 and RRM3, which are all needed for the stabilization of 7SK snRNA and formation of large complex [52-54]. Interestingly, its C-terminus is often deleted in human tumors suggesting that activation of P-TEFb mediated by LARP7 destabilization of large complex is important for proliferation and tumorigenicity of cancer cells. Indeed, RNAi mediated down-regulation of LARP7 blocking mammary epithelial cell differentiation [53]. In the same line, genetic inactivation of MXC, the Drosophila homologue of LARP7, resulted in overgrowth of lymph glands and hematocyte overproliferation [143]. Moreover, down-regulation of LARP7 was reported to be a suitable prognostic marker to predict the presence of lymph node metastasis in early stage of squamous cell cervical cancer before treatment [144]. In conclusion, it is likely that ectopic activation of P-TEFb in cancer cells serves to support transcription of key tumor-progressing genes, unchecked proliferation ultimately converging in tumorigenesis.

## Frontiers

What is the future of P-TEFb in development? We know that targeted inactivation of cyclin T2 in mouse caused embryonic lethality even prior implantation [36]. To be able to follow function of CycT2 in other developmental processes, conditional mouse for CycT2 must be generated first. In these animals, expression of CycT2 can be switch off at a precise developmental stage; then, impact of CycT2 ablation on particular developmental program can be examined. Not only the aspect of CycT2 in early development can be addressed, but function of CycT2 in cell differentiation, regeneration and cell cycle can be revealed, too. Similarly, generation of conditional transgenic mice for other components of P-TEFb complexes will be instrumental to understand their function in various developmental processes.

By utilizing those conditional animals, other important question rendering function of P-TEFb can be revealed. For example, how is P-TEFb recruited to developmental genes? Is recruitment of P-TEFb by a specific activator sufficient or are additional co-factors needed? If so, are these co-factors components of histone-remodeling machinery, mediator complexes? And finally, is P-TEFb released from the large complex and then recruited to the promoter or is large complex bound to promoter/enhancer structures and active P-TEFb is released locally then? Thus, future studies are necessary to gain more light on these and other P-TEFb challenges.

If one thinks of function of P-TEFb in cancer, two aspects of involvement just come to mind. In the literature, one can find examples of indirect or direct involvement of dysregulated activity of P-TEFb in cancer. Down-regulation of Hexim1 and/or LARP7 in breast and cervical cancer most likely leads to increase of free-pool P-TEFb and consequent activation of cancer-related genes. Nevertheless, is simple increase in P-TEFb activity enough to promote malignant transformation or is it part of multi-step tumorigenic process? Yes, activation of P-TEFb in breast cancer cells seemed to be sufficient to promote malignant transformation [53]. Yet, in case of acute childhood leukemia, simple activation has no effect on induction of tumorigenesis but rather misplaced recruitment of P-TEFb through oncogenic chimeric proteins (MLL-ENL) is the driving force [140]. Also, ectopic expression of cyclin T1 in heart did not lead to tumor formation, implying existence of buffering mechanism in cells to deal with elevated P-TEFb activity. On the other hand, ectopic expression of CycT1 in other cellular systems might have deleterious consequences, because of no existing compensatory mechanisms. Again, generation of conditional mouse model might be helpful in this regard to ultimately dissect this conundrum.

**Figure 4:**
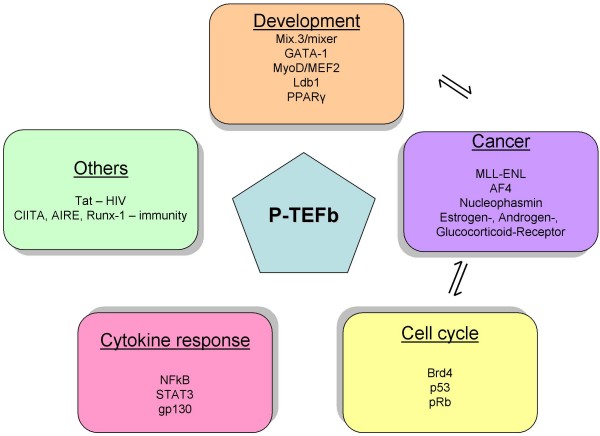
**Role of P-TEFb in a broad spectrum of biological processes**. P-TEFb (blue pentagon) participates in many different biological processes, such as development (light orange oval), cancer (violet oval), cell cycle (yellow oval), cytokine response (pink oval) and others (green oval). Abbreviations in each oval represent particular transcription factors which have been found to employ P-TEFb in given biological phenomena (more information in the text). Importantly, dysregulation of P-TEFb-dependent transcription factors involved in development or cell cycle could also significantly contribute to malignant transformation of normal cells, as depicted by arrows in this figure.

It is becoming increasingly clear that P-TEFb participates in broad spectrum of biological processes (Figure 4). It is feasible to assume that the same transcription factors, different co-factors and signaling pathways co-operating with P-TEFb in cell cycle and development will be involved in pathophysiological effects of P-TEFb in various diseases, too. Therefore, the final task will be to identify what differences in these regulatory circuits are. What is the nature of these changes and if there is a way, to revert de-repressed P-TEFb activity back to normal "physiological state".

## Competing interests

The author declares that they have no competing interests.
